# Using Recombinant Proteins from *Lutzomyia longipalpis* Saliva to Estimate Human Vector Exposure in Visceral Leishmaniasis Endemic Areas

**DOI:** 10.1371/journal.pntd.0000649

**Published:** 2010-03-23

**Authors:** Ana Paula Souza, Bruno Bezerril Andrade, Dorlene Aquino, Petter Entringer, José Carlos Miranda, Ruan Alcantara, Daniel Ruiz, Manuel Soto, Clarissa R. Teixeira, Jesus G. Valenzuela, Camila Indiani de Oliveira, Cláudia Ida Brodskyn, Manoel Barral-Netto, Aldina Barral

**Affiliations:** 1 Centro de Pesquisas Gonçalo Moniz (Fundação Oswaldo Cruz – FIOCRUZ), Salvador, Brazil; 2 Departamento de Enfermagem, Universidade Federal do Maranhão, São Luis, Brazil; 3 Centro de Biología Molecular Severo Ochoa (CSIC-UAM), Departamento de Biología Molecular, Universidad Autónoma de Madrid, Madrid, Spain; 4 Vector Molecular Biology Section, Laboratory of Malaria and Vector Research, National Institute of Allergy and Infectious Diseases, National Institutes of Health, Rockville, Maryland, United States of America; 5 Instituto de Investigação em Imunologia, Instituto Nacional de Ciência e Tecnologia (INCT), São Paulo, Brazil; Institut Pasteur, France

## Abstract

**Background:**

*Leishmania* is transmitted by female sand flies and deposited together with saliva, which contains a vast repertoire of pharmacologically active molecules that contribute to the establishment of the infection. The exposure to vector saliva induces an immune response against its components that can be used as a marker of exposure to the vector. Performing large-scale serological studies to detect vector exposure has been limited by the difficulty in obtaining sand fly saliva. Here, we validate the use of two sand fly salivary recombinant proteins as markers for vector exposure.

**Methodology/principal findings:**

ELISA was used to screen human sera, collected in an area endemic for visceral leishmaniasis, against the salivary gland sonicate (SGS) or two recombinant proteins (rLJM11 and rLJM17) from *Lutzomyia longipalpis* saliva. Antibody levels before and after SGS seroconversion (n = 26) were compared using the Wilcoxon signed rank paired test. Human sera from an area endemic for VL which recognize *Lu. longipalpis* saliva in ELISA also recognize a combination of rLJM17 and rLJM11. We then extended the analysis to include 40 sera from individuals who were seropositive and 40 seronegative to *Lu. longipalpis* SGS. Each recombinant protein was able to detect anti-saliva seroconversion, whereas the two proteins combined increased the detection significantly. Additionally, we evaluated the specificity of the anti-*Lu. longipalpis* response by testing 40 sera positive to *Lutzomyia intermedia* SGS, and very limited (2/40) cross-reactivity was observed. Receiver-operator characteristics (ROC) curve analysis was used to identify the effectiveness of these proteins for the prediction of anti-SGS positivity. These ROC curves evidenced the superior performance of rLJM17+rLJM11. Predicted threshold levels were confirmed for rLJM17+rLJM11 using a large panel of 1,077 serum samples.

**Conclusion:**

Our results show the possibility of substituting *Lu. longipalpis* SGS for two recombinant proteins, LJM17 and LJM11, in order to probe for vector exposure in individuals residing in endemic areas.

## Author Summary

During the blood meal, female sand flies (insects that transmit the parasite *Leishmania*) inject saliva containing a large variety of molecules with different pharmacological activities that facilitate the acquisition of blood. These molecules can induce the production of anti-saliva antibodies, which can then be used as markers for insect (vector) biting or exposure. Epidemiological studies using sand fly salivary gland sonicate as antigens are hampered by the difficulty of obtaining large amounts of salivary glands. In the present study, we have investigated the use of two salivary recombinant proteins from the sand fly *Lutzomyia longipalpis*, considered the main vector of visceral leishmaniasis, as an alternative method for screening of exposure to the sand fly. We primarily tested the suitability of using the recombinant proteins to estimate positive anti-saliva ELISA test in small sets of serum samples. Further, we validated the assay in a large sample of 1,077 individuals from an epidemiological survey in a second area endemic for visceral leishmaniasis. Our findings indicate that these proteins represent a promising epidemiological tool that can aid in implementing control measures against leishmaniasis.

## Introduction

The Leishmaniasis is a widely distributed disease, caused by *Leishmania* protozoans and transmitted by sand fly vectors. Infected sand flies inject parasites when attempting to take a blood meal. In this process, vector saliva is inoculated together with *Leishmania* into the host skin. This saliva is composed of molecules that modulate the host's hemostatic, inflammatory and immune responses [1]. Some of these molecules are immunogenic and stimulate strong immune responses in animals including humans [2],[3]. Importantly, the humoral response against sand fly saliva has been proposed as a potential epidemiological marker of vector exposure in endemic areas of Leishmaniasis [4],[5].

Sand fly populations tend to be clustered [5] leading to unequal exposure of human populations. Screening of human antibodies to sand fly saliva could be a useful indicator of the spatial distribution of sand flies in a particular region. Pinpointing areas of high exposure to sand fly bites may be helpful in directing control measures against Leishmaniasis.

Large-scale serological studies to detect vector exposure have been limited by the difficulty in obtaining large amounts of saliva. Additionally, the use of salivary gland sonicate inherits the limitation of potentially considerable variability in stocks of sand fly saliva due to differences in the feeding source and time of collection after feeding [6]. Salivary protein content varies along the feeding cycle and is influenced by the source of feeding used by sand flies [6].

Another limitation of using SGS is a potential lack of specificity of the salivary proteins due to immunogenicity of proteins present in different species. The use recombinant proteins may reduce such a problem by using proteins which exhibit predominant species-specificity.

Two recombinant molecules, rLJM17 and rLJM11, from *Lutzomyia longipalpis* saliva, were recognized by sera of men, dogs and foxes from endemic areas for visceral Leishmaniasis (Teixeira *et al*., unpublished data), represent good candidates for large scale testing of human exposure to *Lu. longipalpis* bites. In this study, we tested a large cohort for exposure to *Lu. longipalpis*, and validated the results obtained using the recombinant proteins with total sand fly saliva.

## Methods

### Ethics Statement

Informed consents were obtained from all participants and all clinical investigations were conducted according to the principles expressed in the Declaration of Helsinki. The project was approved by the institutional review board from Centro de Pesquisas Gonçalo Moniz-FIOCRUZ/BA.

### Sand Flies and Preparation of SGS

*Lutzomyia longipalpis*, Cavunge strain, were reared at Centro de Pesquisas Gonçalo Moniz-FIOCRUZ, as described elsewhere [7]. Salivary glands were stored in groups of 20 pairs in 20 µl NaCl (150 mM) Hepes buffer (10 mM, pH 7.4), at −70°C. Immediately before use, salivary glands were disrupted by ultrasonication. Tubes were centrifuged at 10,000×g for 2 min and the resultant supernatant (Salivary Gland Sonicate - SGS) was used for the studies.

### Study Design and Participants

**Figure 1:**
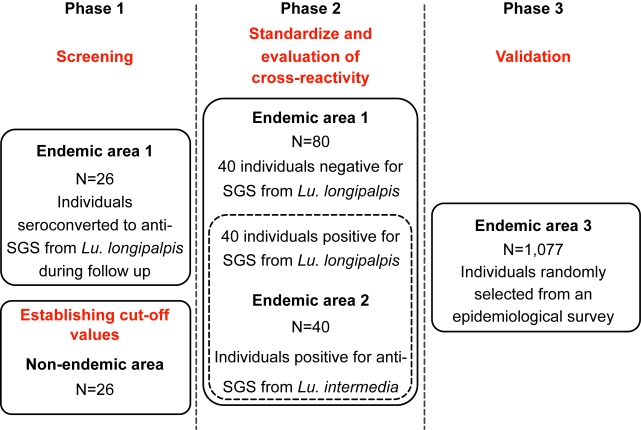
Study flow chart. The study was divided in three phases, each of them using different sample sets to address the suitability for the use of *Lu. longipalpis* salivary recombinant proteins as marker of human vector exposure. To establish the cut-off values form the first phase, 26 indiduals from a non-endemic area were tested for SGS and the recombinant proteins (see details in the methods section). In the first and second phases, samples were obtained from São Luis, Maranhão State, in northeastern Brazil, where VL is endemic and *Lu. longipalpis* is prevalent. To assess cross-reactivity, 40 samples from the VL endemic area who were positive for *Lu. longipalpis* anti-SGS were tested fom anti-SGS from *Lu. intermedia*, while other 40 samples from an endemic area of cutaneous leishmaniasis were used (Canoas, a rural village in Bahia, Brazil) were tested for anti-SGS from *Lu. longipalpis* and the recombinant proteins (dashed line box). The third phase used serum samples obtained from children residing in two other endemic areas for visceral leishmaniasis (Vila Nova and Bom Viver), in Raposa county, Maranhão State, Brazil. The study design details are described in methods. SGS: salivary gland sonitate; VL: visceral leishmaniasis.

This study was divided in three phases (Figure 1). Different sets of serum samples were randomly selected from three independent epidemiological surveys previously performed in two endemic areas for Leishmaniasis (one for VL and the other for cutaneous Leishmaniasis). The selection criteria of the subjects enrolled in each survey are published elsewhere [5],[8],[9]. In the first phase, 26 serum samples were obtained from an epidemiological survey of VL in children less than 7 years old living in a region of São Luis, Maranhão State, in northeastern Brazil, where VL is endemic and *Lu. longipalpis* is prevalent [5]. These samples were selected based on presenting seroconversion against the *Lu. longipalpis* SGS after a follow up period of six months. The cut off value of the anti-SGS ELISA was established as the mean plus three standard deviations (SD) of the mean optical density (OD) of serum samples of 26 individuals from an urban non-endemic area for both human Leishmaniasis and *Lu. longipalpis*. Serum samples with OD above this cut-off (0.073) were considered SGS-positive. The same methodology was applied to assess the cut-off values for the recombinant proteins. The objective was to primary set the cut-off values and to verify the concordance of seroconversion against the SGS and the recombinant proteins.

In the second part of the study, we attempted to check if the recombinant proteins were useful to discriminate anti-SGS positivity. To do this, we randomly selected another 80 individuals from the same endemic area, 40 being positive and 40 being negative for anti-SGS, and performed serology against the recombinant proteins. Receiver-Operator Characteristic (ROC) curves were built for each protein separately and for the combination of both. New cut-offs combining highest sensitivity and specificity and the highest likelihood ratio for this discrimination were determined based on the ROC curves. The ROC curves lead us to identify the effectiveness of these proteins for the identification of anti-SGS positivity.

In addition, to evaluate specificity regarding reactivity to other sand flies, we used 40 serum samples obtained from an epidemiological survey conducted in a region endemic for American cutaneous Leishmaniasis (Canoa, a rural village, located near Santo Amaro, Bahia, Brazil). In this region, *Lu. intermedia* represents the major sand fly species, with *L. braziliensis* being the main *Leishmania* species in the area. Both *Lu. longipalpis* and *Lu. intermedia* normally live in different ecosystems and only rarely individuals are exposed to both of them. Details of the area, patients and anti-*Leishmania* delayed type hypersensitivity skin test are described elsewhere [8]. We used data from 40 individuals exposed to *Lu. intermedia* in a previous investigation [9] and addressed the cross-reactivity to the whole SGS or the recombinant salivary proteins from *Lu. longipalpis*.

The third part of the study was the validation of the serology for detection of antibodies against the *Lu. longipalpis* salivary recombinant proteins as a marker of vector exposure. We used a larger panel consisting of 1,077 sera from another population survey done through home visits. Therefore, serum samples were obtained from children residing in two endemic areas for visceral Leishmaniasis (Vila Nova and Bom Viver), in Raposa county, Maranhão State, Brazil. Vila Nova and Bom Viver have an approximate population of 2,600 and 4,307 inhabitants, respectively. Within this population, a total of 1,297 children under 10 years old were identified and, of these, 1,077 children were enrolled in the study (220 individuals withdrawn consent). The flow chart of the third phase of the study is illustrated in Figure S1.

### Expression and HPLC Purification of His-tagged *Lu. longipalpis* Salivary Proteins

Antibodies to sand fly saliva from endemic area humans, dogs, or fox sera recognize mostly salivary proteins in the range of 15 to 65 kDa. Based on this information we selected nine transcripts coding for salivary proteins from *Lu. longipalpis* falling in this molecular range (LJM17 [AF132518], LJM111 [DQ192488], LJM11 [AY445935], LJL143 [AY445936], LJL13 [AF420274], LJL23 [AF131933], LJM04 [AAD32197.1], LJL138 [AY455916], and LJL11 [AF132510]) [10]. From the range of recombinat salivary proteins tested rLJM17 and rLJM11 were the best candidates recognized by sera from all three hosts (Teixeira *et al.* unpublished data). Recombinant proteins were produced by transfecting 293-F cells (Invitrogen) with plasmids (VR2001-TOPO) coding for these different salivary proteins following the manufacturer's recommendations (Teixeira *et al*. unpublished data). The concentrated supernatant was added to a HiTrap chelating HP column (GE Healthcare) that was then connected to a Summit station HPLC system (Dionex, Sunnyvale, CA) consisting of a P680 HPLC pump and a PDA-100 detector.

### Serology

ELISA was performed as described before [4]. Briefly, ELISA plates were coated with *Lu. longipalpis* SGS, equivalent to 5 pairs of salivary glands/mL (approximately 5 ug protein/mL), or with 1 ug of each recombinant protein/mL (when used independently or in combination) in carbonate buffer (NaHCO_3_ 0.45 M, Na_2_CO_3_ 0.02 M, pH 9.6) overnight at 4°C. After three washes with PBS- 0.05% Tween, the plates were blocked for 1 hour at 37°C with PBS-0.1% Tween plus 0.05% BSA. Sera were diluted 1∶50 with PBS-0.05% Tween and incubated overnight at 4°c. After further washings, the wells were incubated with alkaline-phosphatase-conjugated anti-human IgG (Sigma, Sr. Louis, MO) at a 1∶1,000 dilution for 45 minutes at 37°c. Following another washing cycle, the color was developed for 30 minutes with a chromogenic solution of p-nitrophenylphosphate in sodium carbonate buffer pH 9.6 with lmg/mL of MgCI2.

The concentrations of saliva or recombinant proteins used were determined in a dose- response experiment to assess the optimum signal without loosing specificity. In all experiments, values obtained were subtracted from those obtained the background (i.e. OD values observed in well with only buffer and without SGS or recombinant antigens). The serological experiments were repeated twice with similar results. The laboratory personnel who performed the assays using the recombinant proteins were blinded about the results of ELISA assays for anti-SGS.

### Statistical Analysis

The statistical analyzes were performed using the GraphPad prism software 5.0 (GraphPad Prism Inc., San Diego, CA). Data regarding antibody levels before and after SGS seroconversion were compared using the Wilcoxon signed rank paired test. Kruskal Wallis with Dunn's multiple comparisons test was performed to estimate differences of OD values between three or more groups. ROC curves were used to establish the cut-off values based on the identification of the serology value, which presented the highest sensitivity and specificity in the prediction of anti-SGS positivity. Correlations between the antibody titers against SGS and those against rLJM17 and rLJM11 recombinant proteins were checked using the non-parametric Spearman test. For the validation of the serology in the third phase of the study, the calculation of sensitivity, specificity and predictive values were done through contingence tables. In all instances, differences presenting p<0.05 were considered statistically significant.

## Results

**Figure 2:**
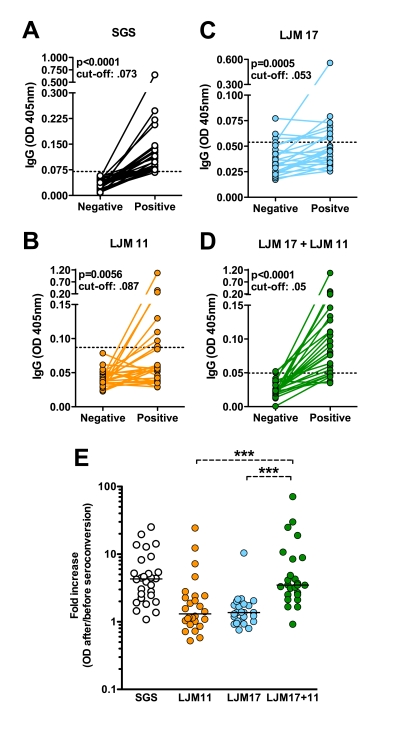
Recombinant *Lu. longipalpis* salivary proteins as a suitable marker of vector sand fly exposure. Sera from individuals residing in a visceral leishmaniasis endemic area (n = 26) were tested by ELISA against SGS, rLJM11, rLJM17 and rLJM11+rLJM17. A, anti-SGS antiboby titers in individuals before and after anti-SGS seroconversion (cut-off value: 0.06). B, anti-LJM 11 antibody levels before and after seroconversion against total SGS (cut-off: 0.067). C, anti-LJM 17 antibody levels before and after anti-SGS seroconversion (cut-off: 0.042). D, antibody levels against rLJM11+rLJM 17 before and after anti-SGS seroconversion (cut-off: 0.038). E, for each individual, the magnitude of the seroconversion was estimated by ratios between the antibody levels after and before the seroconversion. Data regarding antibody levels before and after SGS seroconversion were compared using the Wilcoxon signed rank paired test. The ratios were compared using Kruskal Wallis Test with Dunn's multiple comparisons. Median values from the groups SGS and LJM17+11 were not statistically different. In addition, LJM11 and LJM17 groups were not different but both displayed significantly lower ratios than SGS group (p<0.0001 for both comparisons). ***p<0.0001.

In the first part of this study, we tested whether rLJM17 and rLJM11 are associated with *Lu. longipalpis* exposure we measured the reactivity of total SGS, rLJM17, rLJM11 using serum samples from 26 children that seroconverted to SGS-positive in a period of six months. Figure 2A shows anti-SGS antibody levels at time 0 and 6 months, demonstrating a significant increase in the optical density of the samples. Using the same serum samples, assays were then performed with rLJM17, rLJM11 or a combination of both proteins as antigens. Both rLJM17 and rLJM11 were able to reflect the SGS-seroconversion in a variable number of samples (Figure 2B–C).

Combining both proteins considerably increased the detection of anti-SGS seroconversion, as only four samples were negative within those positive for anti-SGS (Figure 2D). In addition, in agreement with these findings, the OD values fold increases were lower for both anti-rLJM11 and anti-rLJM17 compared with anti-SGS (Figure 2E). The serology using the combination of the recombinant proteins displayed a higher fold increase, similar to the pattern observed for anti-SGS (Figure 2E). Western blot analysis performed for a small number of sera showed that some samples that recognize LJM17 do not recognize LJM11 and vice versa (data not shown; Teixeira *et al*. unpublished data), reinforcing the use of combined antigens to enhance sensitivity.

**Figure 3:**
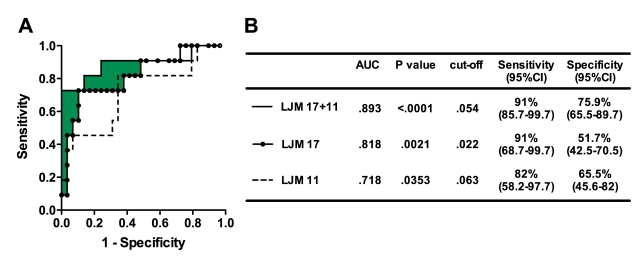
ROC curves of antibody threshold levels predicting ELISA positivity against SGS. A, ROC curves were built using data regarding the serum antibody levels (OD) against the different recombinant salivary proteins obtained from 40 individuals seroconverted and 40 who did not seroconverted according to the cut-off levels described in Figure 1. The filled area represents the increment in the area under the curve from results obtained using rLJM17+rLJM11 compared with the best test using either antigen alone. Samples were tested in duplicate. B, Detailed information obteined from each ROC curve is shown, such as area under curves (AUC), p values of the ROC curves, the cut-off values chosen, and sensitivity and specificity with the 95% confidence interval (CI).

We further evaluated the effectiveness of the recombinant proteins in predicting anti-SGS seroconversion using a larger sample of individuals (n = 80), in which 40 were negative and 40 were positive for anti-SGS. The combination of both rLJM17 and rLJM11 as antigens incremented effectiveness by 8% compared to rLJM17 tested alone and by 17% compared to rLJM11, estimated by the area under the curves (Figure 3A). Thus, serology using these two combined salivary antigens is suitable to discriminate individuals exposed to *Lu. longipalpis* saliva (AUC: 0.89; p<0.0001; cut-off 0.054; likelihood ratio: 8.34) compared with the use of LJM17 (AUC: 0.81; p<0.0001; cut-off: 0.022; likelihood ratio: 5.69) or LJM11 (AUC: 0.72; p = 0.035; cut-off: 0.063; likelihood ratio: 2.16) separately (Figure 3B).

**Figure 4:**
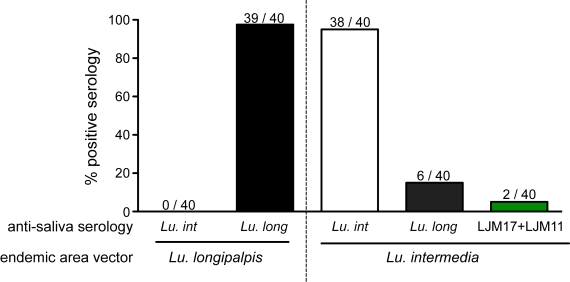
Assessment of the specificity of the *Lu. longipalpis* SGS and the recombinant salivary proteins. A, samples from 40 individuals from an endemic area for *Lu. longipalis* where tested for serology against the *Lu. intermedia* SGS. In addition, 40 individuals from an endemic area for *Lu. intermedia* were tested for serology against *Lu. longipalpis* SGS.

Before SGS or the salivary recombinant proteins could be validated as markers of exposure to *Lu. longipalpis*, it was necessary to assess the specificity by evaluating reactivity towards individuals exposed to other sand flies. Hence, we tested serum samples from an endemic area for cutaneous Leishmaniasis (Canoa, Bahia, Brazil), in which the major species of sand flies is the *Lutzomyia intermedia*. Both *Lu. longipalpis* and *Lu. intermedia* normally live in different ecosystems and only rarely individuals are exposed to both of them. We used data from 40 individuals exposed to Lu. intermedia in a previous investigation [9] and addressed the cross-reactivity to the whole SGS or rLJM17 and rLJM11 from *Lu. longipalpis*. Furthermore, 40 individuals who lived in an endemic area for Lu. longipalpis were tested for anti-SGS from *Lu. intermedia* serology (Figure 4). Almost all individuals from the *Lu. longipalpis* endemic area presented positive serology for this vector, but none of them were positive for anti-SGS for *Lu. intermedia* (Figure 4). Thirty-eight out of 40 individuals from *Lu. intermedia* endemic area displayed positive serology for the saliva of this vector, six also recognized *Lu. longipalpis* SGS (Figure 4), one recognized rLJM11, two recognized rLJM17 and the same two recognized the combination of the two recombinant proteins. Thus, to the end of the second phase of the study we found that both SGS and the recombinant salivary proteins present very low cross-reactivity against a different and wide distributed sand fly. In addition, we established the combination of the recombinant proteins as a potential good predictor of exposure to *Lu. longipalpis*, since ROC curve analysis showed a sensitivity of 91% and a specificity of 76% with the cut-off value of 0.054 OD, with a likelihood ratio of 8.34.

**Figure 5:**
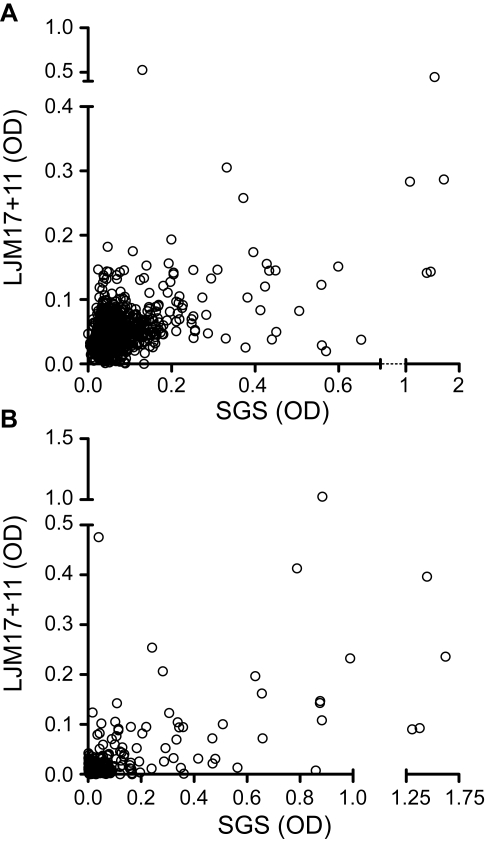
Correlations between antibody production against the whoke saliva and the combined recombinant proteins LJM11 and LJM17. A, Antibody levels (OD) were correlated within 1,077 individuals from an endemic area of viceral leishmaniasis. B, The same correlation was performed in the subgroup of 200 individuals from the 1,077, who seroconverted for anti-SGS during the follow up. Data was analyzed using Spearman test. The values of r and p are plotted in each graph.

**Figure 6:**
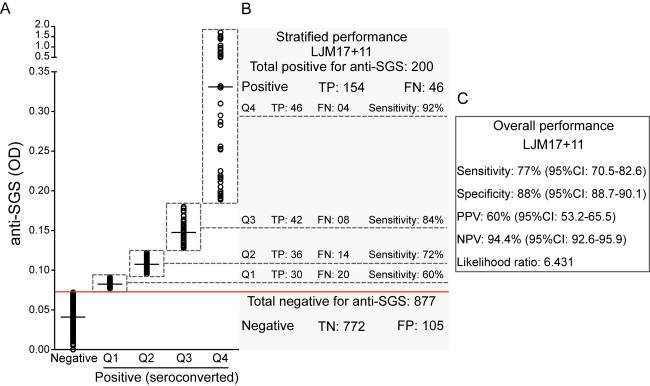
Validation of the combination of rLMJ 17 and rLJM 11 for the estimation of human exposure to *Lu. longipalpis* saliva. A total of 1,077 individuals from an endemic are of visceral leishmaniasis were tested for anti-SGS and for the combined recombinant proteins (rLJM17 + rLJM11). A, anti-SGS titers (OD). The antibodies concentrations of the positive samples were gouped into quartiles (Q). B and C, the overall performance of the serology using the recombinant proteins predicting anti-SGS positivity was estimated, and also the sensitivity of the assay according to the quartiles in positive samples. TP, true positive; FP, false positive; TN, true negative; FN, false negative; PPV, positive predictive value; NPV, negative predictive value; CI, confidence interval.

The final step was to validate the use of these salivary antigens as a reliable marker of exposure to *Lu. longipalpis*. To do so, we tested a panel of 1,077 samples of unknown anti-SGS status from children from another visceral Leishmaniasis endemic area. Sera positive against rLJM17+rLJM11 displayed a positive correlation with anti-SGS IgG levels (Spearman r = 0.379, p<0.0001; Figure 5A). Additionally, when considering only individuals who seroconverted to SGS (n = 200), this correlation became stronger (Spearman r = 0.491, p<0.0001; Figure 5B). The overall performance of the serology using the combined recombinant proteins was satisfactory, with a sensitivity of 77% (95% CI: 70.5–82.6), a specificity of 88% (95% CI: 85.7–90.1), a positive predictive value of 60% (95% CI: 53.2–65.5), a negative predictive value of 94.4% (95% CI: 92.6–95.9), and a likelihood ratio of 6.43 (Figure 6). We then stratified SGS positive cases in quartiles according to optical density values in order to verify if the efficiency of the serology would increment in those individuals with higher antibody titers against SGS (Figure 6A). Concordant and discordant results from the combined rLJM17 and rLJM 11 serology were calculated for each quartile (Figure 6B). The assay using combined salivary antigens presented a general trend with increased effectiveness of prediction in individuals with higher anti-SGS antibody titers (Figure 6B). Thus, the use of the recombinant salivary proteins was effective in the estimation of exposure to the *Lu. longipalpis* saliva (Figure 6C).

Age could be an important factor influencing exposure in the endemic areas and thus we tested whether the concentrations of anti-SGS antibodies were influenced by age. In the age range included in the study, there was no difference in the age distribution of positive individuals within stratified quartiles, (Kruskal Wallis test p = 0.065), indicating that the antibody titers may likely represent exposure to the sand fly.

## Discussion

Antibodies against the salivary gland components of blood sucking insects [11],[12], can be used as epidemiological markers of vector exposure [13], as has been shown for Leishmaniasis [4],[5]. Large epidemiological investigations using salivary gland antigens are hampered by the limitation of obtaining large amounts of highly reproducible salivary glands sonicate. Herein we report on the detection of sera reactive to whole SGS using two recombinant proteins from *Lu. longipalpis* saliva, rLJM17 and rLJM11 and show a positive correlation between the results obtained using SGS and those obtained using rLJM17+rLJM11 as antigens.

Besides the possibility of being produced in large amounts, recombinant salivary proteins bring another advantage to serological tests as they can produce in a highly reproducible fashion. It is known that sand fly saliva protein profile, as well as its relative content, varies at different stages after a meal [6],[14],[15], and this cannot be totally controlled even in standardized colonies.

The combined use of different recombinant proteins is justified since not all SGS-positive sera recognize the same protein bands [5]. In the present study, some serum samples recognized either rLJM17 or rLJM11 when tested by ELISA and this was further confirmed by western blot (data not shown). Such differential recognition may explain the better performance of the test when samples highly reactive to SGS were employed. Other immunogenic salivary proteins are likely candidates to be tested in conjunction with rLJM17 and rLJM11, which may increase the test sensitivity.

Importantly, recombinant molecules selected for use in serology should not cross-react with salivary proteins from other non-vector sand fly species, which may lead to false positive results. In order to test for specificity, we have evaluated sera from one area where *Lu. longipalpis* is highly predominant to one area where this species is not found. In a large survey in the whole São Luis island, including the three municipalities which comprise areas 1 and 3 of the present report, with the capture of 22,581 specimens *Lu. longipalpis* (66.4%) of the captured specimens. It was followed by *Lutzomyia whitmani* (24%) and *Lutzomyia evandroi* (5.9%), with the remaining 29 species represented 3.7% of the total sample [16]. On the other hand, in the Canoa village (area 2 of the present study) a phlebotomine survey performed at the time of sera collection evidenced a marked predominance of *Lu. intermedia*, representing 94% of the captured specimens, with a small number of *Lutzomyia migonei* and *Lutzomyia (Nyssomyia) sp*. [17]. Comparative sequence analysis of LJM17 from *Lu. longipalpis* and *Lu. intermedia* LJM17-homologue showed some areas of high aminoacid conservancy (Teixeira *et al*. unpublished data). However, cross reactivity with *Lu. longipalpis* SGS was not observed in animals experimentally exposed to *Lu. intermedia* SGS [9]. Likely, the tertiary conformation of the LJM17 protein from *Lu. longipalpis*, is distinct from that of *Lu.intermedia* accounting for the specificity of the assay.

In conclusion, we have shown here that ELISA employing two recombinant proteins derived from *Lu. longipalpis* saliva is a powerful tool for detecting specific exposure to vector sand flies in populations. These proteins represent a promising epidemiological tool that can aid in implementing control measures against Leishmaniasis.

## Supporting Information

Checklist S1 STARD checklist.
(0.12 MB PDF)

Figure S1 STARD flowchart.
(0.02 MB PDF)
