# Supplementary figures and images for: Using Recombinant Proteins from Lutzomyia longipalpis Saliva to Estimate Human Vector Exposure in Visceral Leishmaniasis Endemic Areas
Source: PLoS Negl Trop Dis. 2010 Mar 23;4(3):e649. doi: 10.1371/journal.pntd.0000649 (PMC2843636; doi:10.1371/journal.pntd.0000649)

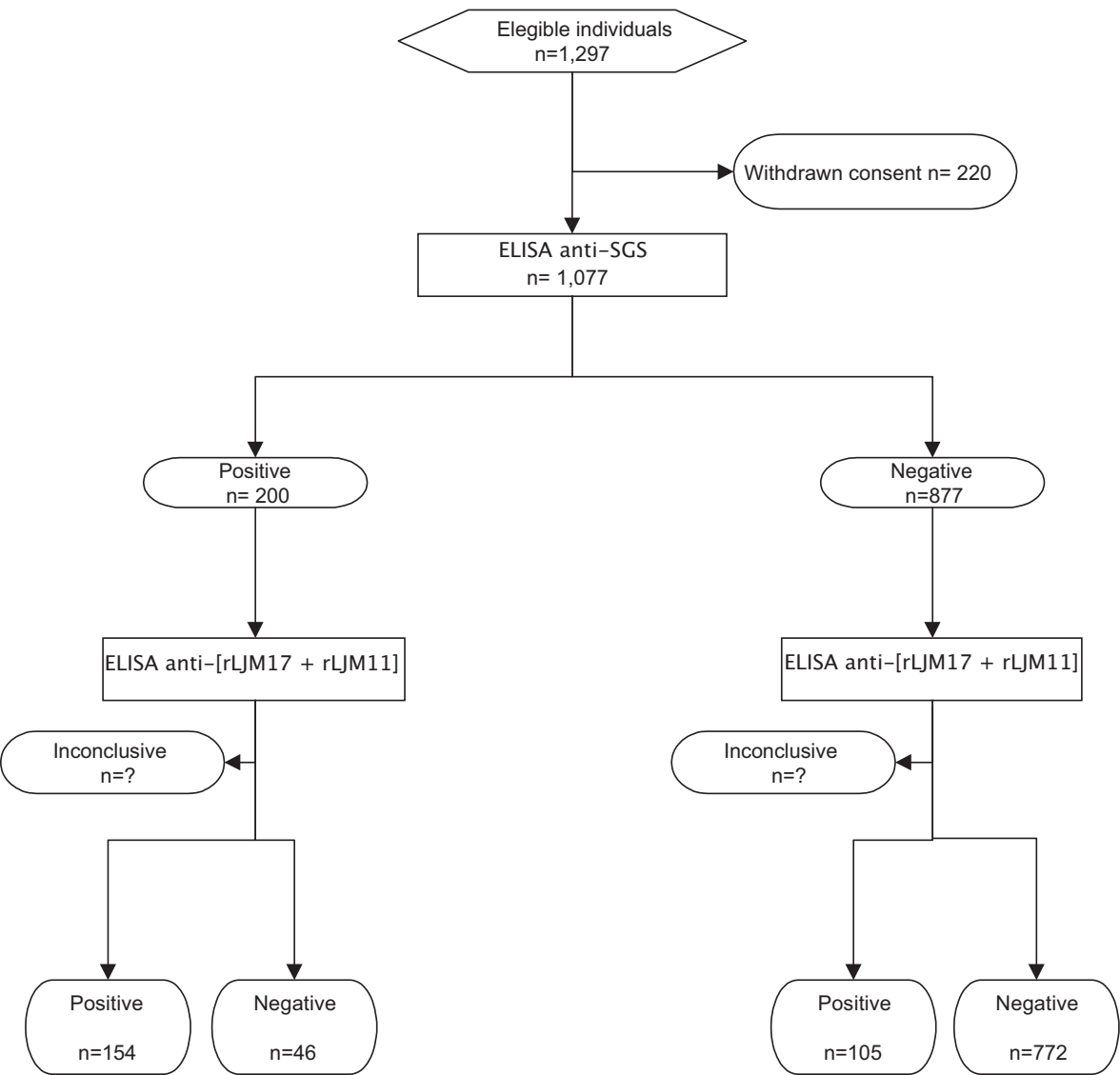

Supplement: Figure S1 — STARD flowchart. (0.02 MB PDF) [file pntd.0000649.s002.pdf]
